# The influence of context on the implementation of integrated palliative care in an academic teaching hospital in South Africa

**DOI:** 10.1177/26323524231219510

**Published:** 2024-01-08

**Authors:** Rene Krause, Liz Gwyther, Jill Olivier

**Affiliations:** Division of Interdisciplinary Palliative Care and Medicine, University of Cape Town, Room 2.28, Falmouth Building, Observatory, Western Cape 7935, South Africa; Division of Interdisciplinary Palliative Care and Medicine, University of Cape Town, Observatory, Western Cape, South Africa; Department of Public Health, University of Cape Town, Rondebosch, Western Cape, South Africa

**Keywords:** context, functional, integration, normative, palliative care

## Abstract

**Background:**

Palliative care (PC) has been integrated to a limited extent in the South African healthcare system. Contextual factors may be a pivotal influence in this integration.

**Objectives:**

This study aims to explore contextual factors that are possibly influencing the integration or lack thereof in an academic teaching hospital (ATH).

**Design:**

A mixed-method study was conducted in a large ATH in South Africa.

**Methods:**

The mixed methods were conducted in parallel and then merged. Findings were integrated to describe the contextual factors influencing PC integration, to develop a timeline of implementation and assess the probable influence of context on the integration process. The mixed-methods phases included a narrative review of published literature related to health systems, integration of health interventions and PC in teaching hospital settings; followed by interviews, documentary and routine data analyses. Semi-structured interviews with purposively sampled participants provided the qualitative data. Primary national, provincial and organizational documents expanded the contextual phenomena and corroborated findings. Routine hospital admission and mortality data was statistically analysed to expand further and corroborate findings. All qualitative data was thematically analysed using deductive coding, drawing from the aspects of the contextual dimensions of integration.

**Results:**

Enabling contextual factors for local PC integration were global and local advocacy, demonstrated need, PC being a human right, as well as the personal experiences of hospital staff. Impeding factors were numerous misconceptions, PC not valued as a healthcare priority, as well as limitations in functional elements necessary for PC integration: national and regional political support, leadership at all levels and sustainable financing.

**Conclusion:**

The normative and functional contextual aspects interplay at macro, meso and micro levels positively and negatively. How stakeholders understand and value PC directly and indirectly impacts on PC integration. Strategic interventions such as mandatory education are required to ensure PC integration. The health system is dynamic, and understanding the context in which the health system functions is core to the integration of PC. This may assist in developing integration strategies to address PC integration and the transferability of these strategies.

## Plain language summary

How the context influences a new palliative care intervention

Palliative care is being integrated in a big hospital in South Africa. However, there are many contextual factors that influence this integration. These factors are both structural aspects like policies, but also how role players’ values and understand of palliative care. It is important to know what these factors are and how these factors influence integration. This study describes the factors and how they influence palliative care integration.

## Introduction

In 2014, South Africa was one of the co-sponsors of the World Health Assembly Resolution 67.19, ‘Strengthening of palliative care as a component of comprehensive care throughout the life course’.^1^ In this resolution, member states committed to integrating palliative care (PC) into their health systems and to domestic funding of PC.^2^ However, despite this commitment, South Africa still lacks the ability to deliver PC to the whole country as a public service.^3^

There is strong evidence that PC can be a cost-saving intervention.^4,5^ It is necessary to achieve Universal Health Coverage,^6^ and according to the World Health Organization (WHO), PC is an ethical responsibility of all health systems.^2^ Support for PC integration was initiated with the approval of South African National Policy Framework and Strategy for Palliative Care (NPFSPC) in April 2017.^1^ It specifically mentioned academic teaching hospitals (ATHs) as academic centres of excellence for PC teaching, service delivery, advocacy and research.^1^ However, this national policy has not yet been fully implemented across the public health service, including ATHs. Financial resources for integrating PC have thus far been limited, thereby contributing to the constraints on integration.^7^ However, literature which describes integration of other complex interventions indicates that contextual factors other than limited resources may be inhibiting implementation. Additionally, the fact that financial resources are limited may also be due to other contextual factors. Contextual factors in integrating any intervention are a crucial dimension of the complexity of the integration process. Furthermore, the integration of complex interventions may pivot around their suitability in a specific context.^8^ This study will assess the contextual factors that potentially influence the integration of PC into an ATH in South Africa as an example of how contextual factors affect PC integration.

The process of integrating a new service or program into a healthcare system is complex and the intervention often fails to be translated into meaningful outcomes,^9,10^ as interventions cannot be fully isolated due to the influence of factors internal and external to a system. Atun *et al.*^10^ hypothesize that there is an interplay between complex facets that influence the integration of interventions into the health system. These facets are the characteristics of the problem, the intervention itself, the adoption system, health system characteristics, and the context.^10^ Furthermore, it is argued that context is pivotal to understanding the complexity of integration and consists of multifaceted aspects.^8^

‘Context’ is not uniformly defined in the literature and requires insight into the intervention to unpack critical contextual factors that might influence the integration process. Certain standard dimensions are known to be important, such as organizational support, financial resources, social relations and support, leadership, organizational culture and climate and organizational readiness to change.^11^ In addition, the patient profile becomes important when evaluating the context of a healthcare system.^11^ Furthermore, timing is critical for understanding how contextual factors merge at a specific time to influence integration.^12^ The human dimension relating to factors such as motivation, vision, organizational culture, education and assumptions add to the complexity of what constitutes context.^12^

PC is recognized internationally as an essential part of ATHs as supported by the World Health Assembly (WHA) 67.19, but the complexity of PC may play a role in challenges to its integration in that context.^2^ Kaasa *et al.*^13^ emphasize that PC should be provided alongside disease-oriented cancer care as part of comprehensive service delivery in ATHs striving for excellence in care, research and teaching. The reality is that PC should be available for all patients with serious health-related illnesses.^14^ But as stated by Atun *et al.*,^15^ the problem itself (the social narrative, the burden and the urgency of the situation around the problem) influences the integration process. PC is a complex evidence-based intervention where bio-psycho-social and spiritual factors are interwoven with clinical factors to manage patients with life-threatening illnesses.^16^ It involves care for patients with complex, incurable diseases, who often experience a heavy burden of complicated symptoms.^14^

Furthermore, the complexity increases because the symptoms are managed using scheduled drugs, such as opioids, which are subject to many misconceptions, such as risk of addiction.^14^ Patients and their families experience psycho-social change and loss when a patient is diagnosed with a severe illness. This is amplified when combined with poverty and limited access to disease-modifying treatments.^17^ This leaves many patients facing death and their families economically and socially vulnerable and hopeless. Managing affective issues is a core part of PC not only among patients and families but for healthcare professionals (HCPs) as well. Feelings of failure and hopelessness are common findings among HCPs working without PC resources when dealing with patients with life-threatening illnesses.^18^ PC is practised in multidisciplinary teams that function closely to manage each situation’s complexity.^16^ It is also best practised across the continuum of care and thus requires care networks to ensure non-abandonment of patients and families.^16,19^ The integration of PC is, therefore, a complex intervention, and when that integration occurs in an ATH, even more so. Thus, successful integration of PC into ATHs, requires an understanding of the particular contextual factors facing implementors.

### The interconnectedness of context

Multiple contextual dimensions are at play when integrating PC in an ATH. Moreover, these dimensions are interconnected and dynamic.^20^ Valentijn *et al.* state that integration must occur across a health system’s micro, meso and macro levels. They also separated these dimensions into the functional and normative elements that need to be developed and maintained to integrate an intervention.^21^ Functional aspects include support functions and activities around the primary process of service delivery to coordinate and support accountability and decision-making between organization and professionals. Normative elements describe the common and shared frame of reference (values, mission, vision and culture).^21^ Furthermore, functional and normative integration ensures continuity of integration across the levels of the health system.^22^ For example, links of service delivery can be ‘vertical’ (bringing together services operating at different levels) or ‘horizontal’ (the linkage of services on the same level).^23^ Practically, the integration may even be ‘oblique’^15^ – which implies that elements of both vertical and horizontal integration may be needed to solve the problem or to attend to the patients’ needs. It is proposed that PC can be a complex oblique intervention across all levels of the health system, from ATH and primary care to home care.^2^ PC is thus a complex, interlinked intervention that will only be successful if the integration happens across the whole health system.

Awareness of these interlinked contextual factors across the health system is core to the integration process and to evaluating what and why things happen. For example, it allows interventions to be transferred between different settings and explain different outcomes. Understanding context also assists in making general statements about interventions; this is especially important when we are integrating a new intervention, such as PC, across a country’s health system. Finally, the importance of understanding context is especially relevant when managing limited resources because it assists in strategically managing interventions to have the most significant impact. Interventions should aim to be responsive and appropriate to the unique context.

### Brief background to the context of PC integration in a South African ATH

There are considerable socioeconomic and health disparities and a unique disease burden in South Africa. Thirty per cent of doctors in South Africa serve 84% of the public healthcare sector population.^24^ HIV, tuberculosis, interpersonal violence and high incidence of non-communicable diseases (NCDs) such as diabetes burden the South African Health sector.^24^ Moreover, South African ATHs are renowned for addressing this unique disease profile with the latest technology and highly specialized skills, conducting cutting-edge research and training future HCPs. ATHs are an essential part of a comprehensive healthcare system and contribute to strengthening the health system.^25–27^ Furthermore, ATHs are centres where research is undertaken, and staff are involved in guidelines and policy development, such as the Standard Treatment Guidelines and Essential Medicine List.^26,28^ HCPs are trained, and the foundation of their attitudes and values in health care are developed in this setting.^28^ Many patients come to ATHs with complex illnesses to consult experts in these illnesses, where many patients are diagnosed with life-threatening diseases. Following this diagnosis, patients must be identified as requiring PC alongside disease-modifying treatment.

### The PC context in an ATH in South Africa

**Figure 1.**
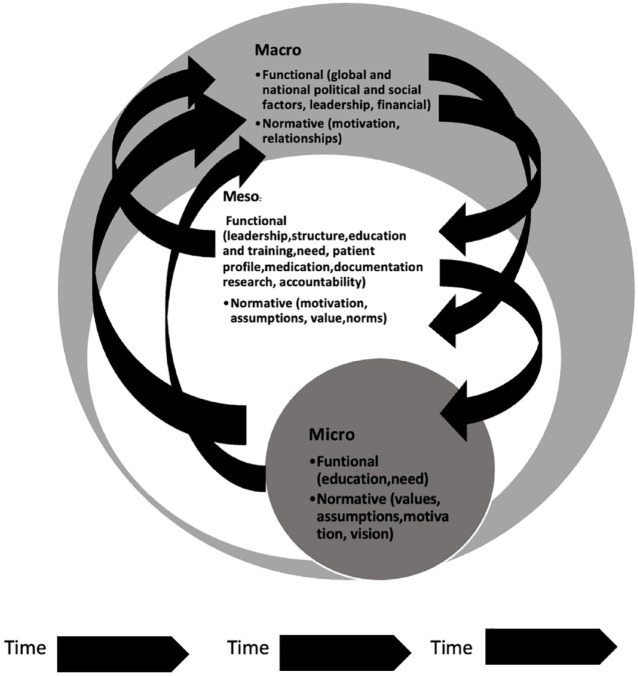
The influence of contextual factors in an ATH (see discussion above). ATH, academic teaching hospitals.

The following dimensions can be considered part of the ‘context’ in an ATH in South Africa (SA)(see Figure 1). Firstly, the macro factors where the global, national and regional political, social, leadership and financial factors constantly change and influence PC and the ATH,^10^ that in turn are influenced by normative aspects such as motivation and relationships.^21^ Secondly, at a meso level, functional aspects such as leadership, structure, education and training, the need, patient profile, availability of medication, documentation, outcome measurements and research interplay with normative aspects.^21,29^ These normative aspects are motivation factors, norms, assumptions and values in the hospital.^21^ Finally, the individual or micro level, describes the values, assumptions, vision and motivation that play a role in PC integration. In addition, functional aspects such as level of training and HCP need also influence the context.^13,29^ All of the above factors are woven together to positively and sometimes negatively affect the implementation of integrated PC, creating a unique lattice in each setting. The uniqueness of this lattice should be considered when evaluating context.

Contextual factors are enablers or barriers, and their dynamic interplay is vital to achieving integration. The aims of this study are to explore potential contextual factors at play in an ATH to explain ‘why’ and ‘how’ PC integration is completed or not achieved, to identify appropriate integration strategies and increase transferability to different settings.

## Methodology

A mixed-methods study was conducted in Groote Schuur Hospital (GSH) in the Western Cape province of South Africa. GSH is a 975-bed ATH with over 3500 personnel. It serves patients in the Western Cape on a pay-as-you-earn basis, but most patients are from impoverished backgrounds and thus pay a nominal fee. Patients are referred to the hospital from primary care settings or from other hospitals in and outside the province and provide tertiary and quaternary care to. In addition, the hospital trains undergraduate and postgraduate HCPs across Africa. The hospital is linked organizationally and administratively to the University of Cape Town.^27^

This mixed-method study integrated quantitative and qualitative data in four phases, begininig with a narrative literature review, followed by three phases conducted in parallel to expand on the contextual phenomena, corroborate and explain the interconnectedness between the different levels: semi-structured interviews (*n* = 8) and analysis of organizational documents (both thematically analysed), and an analysis of quantitative hospital data displayed through descriptive statistics.

The narrative review of published literature in English over the last 20 years entailed searching databases such as CINAHL, MEDLINE, Africa-wide (*via* EBScoHost) and Scopus and Scielo. Materials were gathered within a 2-year framework, and further literature was added, drawing from basic disciplinary research of Palliative Care Research, Health Policy and Systems Research and Integration and Implementation studies.

Qualitative data were collected through semi-structured interviews (*n* = 7) at hospital- and national-levels. At hospital-level, the interviews were conducted with purposively sampled managerial staff from January 2020 until January 2022. The aims were to explore their understanding of PC, the value they accorded it and their experiences of implementation. The managerial staff interviewees were hospital PC executive team inclusive of doctors (*n* = 5), a social worker manager (*n* = 1) and a nursing manager (*n* = 1). These managers represent heads of departments of all the major disciplines and professions involved in implementing PC (surgery, internal medicine, oncology, nursing, social work as well as health and rehabilitation). They are the ‘gatekeepers’ who manage and allocate resources to the PC team in the hospital as a collective decision-making body. The interview guide was developed, drawing on the literature and including aspects of normative and functional integration and contextual dimensions. To enhance rigour, face validity of the interview guide was ensured by presenting the interview guide to experts in the field of integration and the field of PC. Construct validity was derived by aligning this tool with a theoretical integration approach and context. The interview guide was piloted on two managers and did not require adjustment.

At national-level, an interview with the senior national manager in South Africa was conducted in 2021, and explored how the South African PC policy (NPFSPC) came about and how ATHs fit into this national integration. An interview guide was used, drawing from the theory of context. A description of the timeline, leadership, financial matters and history of the national development of PC and how it relates to ATH assisted in providing information about how external political and social factors contribute to the implementation of PC.

The hospital and national-level interviews were thematically analysed using NVIVO (NVivo is a qualitative data analysis computer software package produced by Lumivero). Hospital managers were de-identified and only identified as ‘*Man1*’ etc. The senior national manager is identified as ‘*NM*’. The interviews were analysed using deductive coding for themes of contextual factors at the macro, meso and micro-level (see Figure 1). Inductive coding was used to identify emerging themes. The themes and sub-themes that arose from the analysis of the interviews were coded.^30^ Thematic coding and analysis was checked between all researchers (*n* = 3) and research assistants (*n* = 2).

Quantitative data in the form of the number of hospital admissions, the number of deaths and the number of patients referred to a PC service were obtained from routine admission data from the hospital and Palliative Care Service collected on a RedCap database. This database records routine data on all patients receiving PC from a vertical nurse-led PC service. This research used descriptive data from the database to illustrate the PC integration process from January 2017 until June 2021. The quantitative data were statistically analysed to describe the patient population.

Additional information on the contextual factors that drive integration was drawn from secondary documents describing population, resources, guidelines and standard operating procedures (SOPs) that influence the integration of PC. This was further informed by analysing provincial and national policies on PC. Ten documents were purposively sampled, based on information derived from the interviews. Documentation identified and sourced were the National Policy and Strategic Framework for Palliative Care^1^; the National Cancer Strategic Framework for South Africa (2017–2022)^31^; the National Strategic Plan for the Prevention and Control of Non-communicable Disease (2020–2025)^32^; the National Health Insurance Bill^33^; the National Hospital Level (Adult) Standard Treatment Guideline and Essential Medicine list^34^; the National Tertiary and Quaternary Level Essential Medicine Recommendations^35^; the Proposed Model for the Implementation of the Palliative Care Policy in the Western Cape^36^; the Operational Guide to Implementing Palliative Care during COVID-19^37^; (Provincial) Providing Palliative Care during the COVID-19 pandemic^38^; SOPs that include PC and stationery used in the palliative service used in the hospital.

The documents were analysed thematically, drawing from the themes of integration. The documents are referenced, and hospital documents are listed as hospital documents (HD) in the results. Further document analyses enhanced findings by evaluating authorship, historical insights and factors excluded from the documents.

Finally, the results from the different approaches were merged and compared to identify consistencies or discrepancies across the datasets. These inferences are demonstrated (see Table 1) to explain the context and corroborate findings to gain a better understanding of the phenomena. A timeline describes how the context changes over time, and the impact of global and national PC integration.

**Table 1.** Table of themes identified across data collection methods.

	Functional aspects		Normative aspects		Inference
Macro environment	Policy	• National policy approved in 2017 and provincial model on implementation approved in 2018^1,36^ • PC integrated and aligned in National Health Insurance Bill,^33^ the National Strategic Plan for the Prevention and Control of Non-communicable Disease (2020–2025),^32^ and the National Cancer Strategic Framework for South Africa (2017–2022).^31^ • No M&E mechanisms in place in the NPFSPC^1^ but opioid monitoring mentioned in policy for the Prevention and Control of NCD (2020–2025)^32^ • No funding formula included in the policies but PC mentioned in the proposed NHI bill^33^	Motivation	• The quadruple burden of disease in South Africa^1^ • Advocacy using the principle that PC is a human right (National Co-ordinator and Policy)	There is converging evidence that there is a recognized need for PC but it is not seen as a healthcare priority
	Leadership^1^	• The Minister of Health was committed to implementing the resolution in South Africa^1^ • A national PC coordinator facilitates PC integration and is appointed on a contract (NM) • Provincially a PC task team chaired by a senior provincial director (NM) • PC Task Team developed delivery and Clinical Care guidelines during COVID pandemic^37,38^	Relationships	• Existing relationships in teaching, service delivery and general comradery exist between PC service delivery teams, the universities and national and provincial leaders (NM) • PC is not seen as a healthcare priority (NM)	
	Governance	• No accountability structures included in the national nor provincial policies^1,36^			
	Finance	• PC was historically funded by the NGO sector • Provinces motivate and apply to National Treasury for additional funding (NM)			
Meso environment	Need	• Disease burden and complexity of diseases (database) • Lack of trained PC workforce (Man 5) • Assisting in HCP moral distress when dealing with end-of-life care (Man 3) • Lack of PC services in the district (Man 1 and 3) • Research for policy development (NM)	Motivation	• Driven by personal experience. (NM, Man 2 and Man 4)	There is converging evidence that the hospital value specialized disease-specific care and aim to avoid patient deaths, which can be seen as treatment failures. There’s a shortage of PC-trained HCWs to address misconceptions about PC. These values and assumptions may be the cause of the lack of aspects of functional integration.
	Structure	• Siloed structure across the hospital • Vertical nurse-led PC service (Man 5)	Assumptions	• Refer to PC when there is nothing further the treating team can do • PC is only end-of-life care	
	Education and training	• Four trained professional nurses at intermediate level (Man 5) • Initial 100 HCP trained in PC (Man 5) • Continues bedside mentoring by PC team (Man 5) • Limited engagement in training because current training does not advance career pathways (Man 4)	Values	• Emphasis on curative care and bed turnaround time (Man 2) • PC must be visible on the clinical platform (Man 2)	
	Leadership	• Perceived leadership to integrate PC is with CEO/executive management (Man 4 and 5) • PC service is managed through a distributive leadership (Man 5) • PC service supported by academic leadership from the university (Man 5)	Norms	• Avoidance of end-of-life care • Late referral to PC service (Man 1 and 5)	
	Documentation	• Most SOP’s and guidelines created by PC team • Limited integrated guidelines (only 2 found)			
	Medication	• WHO essential medicine list available^34^			
	Research	• Limited in or with PC team (Man 1–5)			
Micro	Education and training	• Limited training among HCP (Man 5) • Need for basic PC training (Man 5)	Motivation	• Personal experience (Man 2 and 3)	There is converging evidence that moral distress among HCWs is a crucial factor to consider when integrating PC. It can either hinder or facilitate the integration process.
	Need	• Assist in addressing moral distress (Man 2 and 5)	Assumptions	• No further PC training is required (Man 1, 3 and 4) • *But that*: PC is only end-of-life care (Man 4)	
			Vision	• Principles of PC policy supported (Man 3–5)	
			Value	• Discomfort around own mortality (Man 2)	
			Norms	• Dignified end-of-life care (Man 2)	

Data was collected in parallel. Documents collected from the database are indicated by the word ‘database’, data collected from interviews are indicated by interviewee identifier and data collected from documents are indicated by a reference to the specific document.

CEO, chief excutive officer; HCP, healthcare professional; HCW, health care worker; M&E, monitoring and evaluation; NGO, non-government organization; NCD, non-communicable disease; NHI, national health insurance; NPFSPC, National Policy Framework and Strategy for Palliative Care; PC, palliative care; SOP, standard operating procedure; WHO, World Health Organization.

The primary researcher is embedded in the national and local integration process of PC, including the process at GSH. In GSH she contributes to the leadership and evaluation of PC services. A fundamental advantage of being embedded is that the researcher may identify problems that might not be clear from the outside.^39^ Although embedded research does provide further insight; bias may influence the trustworthiness of the findings. The methodological triangulation of data strengthened trustworthiness by asking permission from sources about using information obtained outside the formal research process and sharing results with participants before any publications.^40^ Researcher assumptions were explored upfront using a recorded reflective interview between the researcher and supervisors before the research was conducted. Reflexivity was further ensured by keeping a journal with short notes on discussions by the researcher with the research assistant, who also maintained a journal recording thoughts after the interviews.

## Results

The results in this section come from integrated analysis of all four research phases.

### The PC context at a macro level in South Africa

This section describes ‘functional’ contextual factors which impact on PC integration, namely: global, national, regional political and social factors, leadership, as well as financial factors. As outlined above, they are influenced by normative aspects such as motivation and relationships at a macro level.

The PC National Co-ordinator (NCo) was appointed in 2020 and the post was funded by a donor funder. The incumbent confirmed that the motivations to integrate PC in South Africa were facilitated by global advocacy,^2^ national non-government organization (NGO) advocacy using the advocacy trigger of PC as a human right,^1^ as well as the quadruple burden of disease in South Africa which informed the development of PC policy. South Africa was one of the original sponsors of the WHA Resolution on PC, and the Minister of Health was committed to implementing the Resolution in South Africa.^1^

PC is integrated into the National Health Insurance Bill,^33^ the National Strategic Plan for the Prevention and Control of Non-communicable Disease (2020–2025)^32^ and the National Cancer Strategic Framework for South Africa (2017–2022).^31^ These documents are aligned with the National PC Policy and advocate for PC in the tertiary setting.

There is a need for an integrated, life course approach which includes prevention, education and awareness, screening and early detection, diagnosis and treatment as well as rehabilitation and palliative care. Central to all these interventions is recognising the complex needs of our people, as well as respecting the rights and dignity of patients and their families at all times.^31^

The PC NCo is situated in the NCD directorate in order to facilitate leadership and PC integration across all provinces. In the Western Cape, a senior director has taken the lead as an additional task to lead the provincial Palliative Care Task Team. This task team comprises stakeholders from district healthcare services, hospitals, NGOs, universities and support structures in the provincial government. Currently, two members from GSH contribute to this provincial leadership structure, together with a volunteer specialist from the university.

Accountability and financial support structures for PC are lacking both nationally and provincially.^1,36^ Donors have historically funded PC *via* the NGOs service. According to the senior national manager interviewed (NM), the National Department of Health is responsible for policy-making and administrative duties. Provinces are responsible for applying for funding to the national treasury, and the funds are allocated according to their requests. District coordinators are responsible for motivating for PC funding when provincial budgets are being prepared. Therefore, the provinces carry the bulk of the financing responsibility.

Goal 4 in the national PC policy is to develop a costing formula for PC.^1^ It was intended to be completed by 2018, but to date, there is no national costing formula or funding track. The NM postulates that it is because PC is not perceived as a priority:

Because they feel like palliative care is not a healthcare priority. (NM)

### PC integration in GSH in relation to provincial, national and global PC integration

**Figure 2.**
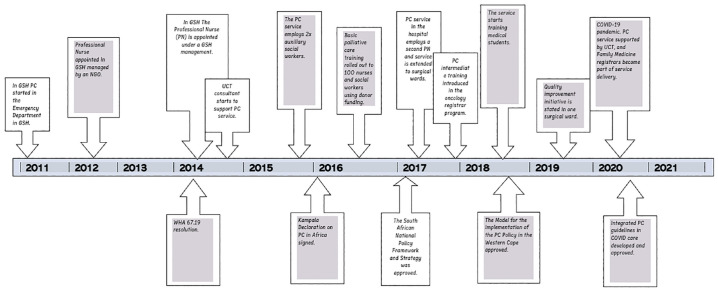
Timeline of PC integration at Groote Schuur Hospital in relation to provincial, national and global PC integration. PC, palliative care.

This section presents a timeline of how PC was integrated in GSH in 2011 and grew, as a result of support from the university, through teaching and training. The construction of the timeline was informed by interviews conducted with the Professional Nurse Manager and NM, as well as a documentary review of the national and provincial policies.^1,36^ The timeline was further validated through the WHA 67.19 Assembly declaration (2014), the Kampala declaration (2016) and the National (2017) and provincial policy (2018) (see Figure 2).

## The PC context at a meso level in South Africa

PC integration at a meso level is influenced by the need for PC; hospital leadership; PC service delivery structure, education and training; patient profile; medication availability; documentation supporting PC; research and their interplay with normative aspects which will be elaborated below.

Table 2 illustrates the need for PC – showing routine hospital data from 2017 to 2021. For example, consider the proportion of deaths in the hospital to number of referrals to PC.The NPFSP argues that 50% of all deaths require PC.^1^ Therefore, we expect a minimum of 3.15% of the admissions to be referred to PC. This was also supported by the interviewees – for example, ‘The need is very big, very, very big’ (Man 2).

**Table 2.** Patient demographics and hospital information.

Hospital information	2017	2018	2019	2020	2021	Overall
Number of PC referrals	737	593	773	931	1195	3585
Number of hospital admissions	50,010	51,504	53,956	44,024	42,206	241,700
Number of deaths in hospital	2825	3027	2741	3133	3549	15,275
Proportion of admissions that died	5.7%	5.9%	5.1%	7.1%	8.4%	6.3%
Proportion of admissions that were PC referral	1.5%	1.2%	1.4%	2.1%	2.8%	1.8%
Proportion of deaths that were PC referral	26.1%	19.6%	28.2%	29.7%	38.4%	27.7%

Source: GSH routine hospital data.

PC, palliative care.

The hospital structure in service delivery and leadership is vertical in all disciplines, with clinical leadership situated with consultants in each discipline who focus on their specific disciplinary needs and structure.

. . .it’s a hospital that is very siloed. In fact, the whole ethos and nature of work in this hospital is that people fight for their own . . . And then there are highly specialised areas who fight against others for resources, but you fight in your own, in your own lane. (Man 4)

Two managers indicated that leadership in the hospital for integrating PC lies with the executive management and or CEO as indicated. There is acknowledgement by three managers that the university plays a major role in providing support in integrating PC.

PC-trained staff are limited to four professional nurses with intermediate-level PC training (Prof Nurse Manager). However, continuous PC bedside mentoring is happening in the hospital (Prof Nurse Manager). It is acknowledged that participating and completing accredited training is contingent on training that will advance careers. (Medical Manager).

For people to do palliative care training, that doesn’t advance their career and for interest, and that. . . no one’s done that. (Man 4)

In clinical disciplines where accredited training has been introduced, there is a recommendation that intermediate-level PC training should be extended to other clinical disciplines (Man 3).

Despite limited trained staff, the presence of a vertical specialized PC service in the wards, which improves access to a PC approach, has increased awareness (Man 2, 4, and 5).

but I think that awarness better now than it was five years ago because there is a much more active, visible presence. (Man 3)

Two integrated hospital PC guidelines were available. The first was a guideline incorporated as an additional chapter in the surgical guideline (authored by the university). The second, authored by the pulmonology team, asked whether patients were referred to PC when receiving home oxygen. Hospital guidelines, authored by the vertical nurse-led service, formulated SOPs for ‘end-of-life’ as well as for ‘syringe drivers’. In addition, they authored documentation on referral criteria, PC assessment and monitoring PC patients. All the PC medications as advised by the WHO essential PC medication, are available in the hospital.

Personal experience seems to be the trigger for involvement in PC in the ATH, as confirmed by three managers and the NCo.

So, it’s their own experience that’s a main motivator for integrating palliative care. (NM)

Three participants commented that there is avoidance of working with PC patients, as well as in the field.

I was talking about it at one of our regional meetings, and one of the heads of surgery in a regional hospital outside, when I said, you know, you need to engage in palliative care processes and thinking, he said ‘I don’t want all those people dying in my ward’. (Man 2)

The assumption that PC is only about end-of-life care among the clinical community was widely recognized by participants.

. . .main assumption was that when you’re referring a patient for palliative care, there’s nothing more that can be done for the patient. And that’s why I think, in many settings, the medical team kind of withdraws from palliative care you know, because they think their job is done. (Man 1)

## The PC context at a micro level in South Africa

As previously outlined, PC integration at the micro or individual level is influenced by the values, assumptions, vision and motivation of individuals. In addition, functional aspects such as level of training and personal need also influence the context.

It is evident from multiple managers that there is an element of moral distress associated with working with patients requiring PC.

I think there’s a, there’s a huge amount of distress. I think, uh, it depends on how close the engagement has been with the family and the patient. Uh, I think there’s distress from the nursing side, the medical staff are often very distressed by patients dying. That triggers a. . . a frequent desire to do stuff, to over-intervene, to check, test and administer things, uh, most of which are harmful and delay the inevitable or, or they are futile attempts that delay the inevitable. (Man 2)

There is an assumption held by two of the participants that PC is only end-of-life care, but all the participants state that this assumption is prevalent in the hospital. The participants value PC, and dignity in death is an important aspect of care (Man 1 and 3). However, there is also avoidance of dealing with their own mortality and medicalizing suffering when faced with PC situations (Man 2–4).

Because. . . maybe there are things they haven’t dealt with themselves about mortality and. . . umm. . . so they will stick to. . . when they explain things to patients, they will use scientific terms and jargon that patients don’t understand because that’s how they feel comfortable. (Man 4)

The managers are aligned with the vertical and horizontal PC services policy (Man 3 and 4), and emphasize the need for strengthening PC services at district-level (Man 3 and 4). There is a vision that all healthcare providers should have a basic understanding of PC (Man 5). However, there is a concern that doctors do not recognize that they need further training.

And what do you think are the biggest assumptions, umm, regarding your discipline and palliative care? The biggest assumption is that consultants know all about palliative care. (Man 4)

## Discussion

This research has demonstrated the complexity of the normative and functional contextual factors and the interconnectedness between all the levels of health care involved in PC integration.^22^ In addition, as Valentijn *et al.*^22^ state, it is the normative aspects that enable the interconnectedness between all the levels of health care, and in PC integration, the normative aspects are core because of the complex and emotive nature of a PC intervention.

### Policy

On a macro level, it is agreed that the NPFSP and global advocacy are facilitators promoting integration of PC into the health system in South Africa. The vision of the policy is supported by managers working with patients requiring PC and who aim to integrate PC. It is further aligned and integrated in other national policies.^31–33^ Therefore, the policy is acceptable in GSH and aligned with a global and national vision.^2^ A shared vision is an essential normative dimension of integration and this vision has been operationalized at a macro level by appointing a national PC coordinator in a leadership role although the position is for a limited time and donor-funded. As highlighted by Valentijn *et al.*,^21^ leadership is an enabler if the leader is senior and formally appointed. Without formally appointed and vocal PC leadership, the PC agenda may not receive the attention required. This is important at all levels, especially when there is a need to advocate for appropriate resource allocation to PC.

### Financial resources

One factor that requires urgent attention is allocating financial resources to PC. Highlighting the need for PC and building on the ‘human rights’ argument, further advocacy is needed to ensure appropriate funding is allocated to promote alignment with the WHA 67.19, the South African NHI Bill and the NPFSPC.^1,2,33^ Unfortunately, to date, limited national funding has been allocated, negatively impacting the meso and micro levels of integration. For example, if there is no funding to provide the care, then the impact of the care cannot be observed and further valued. The lack of funding may be further underpinned by the normative aspect of integration that PC is not valued as a healthcare priority. The fact that South African health authorities are not prioritizing PC further implies lack of value assigned to end-of-life care. A cross-country comparison of experts’ assessments of the quality of death and dying conducted in 2021, demonstrated that South Africa was one of the lowest-ranked (73/81) countries when evaluating the quality of death and dying globally.^41^ This cross-country comparison identified investment in PC and access to affordable PC as aspects that enable better care at the end of life. The poor state of how South Africans die may be linked to the limited PC resources.

### Current situation

Although there are limited national resources being allocated to PC, GSH has developed a PC service in the hospital as a result of advocacy rather than policy, according to five managers. The passionate advocacy based on personal experience by the Emergency unit manager, staff and neurosurgery facilitated necessary resource allocation. Furthermore, this advocacy has contributed to the valuing of a PC approach and identifying the need for a PC service.^42^ In 2014 GSH appointed a PC nurse who provides clinical PC services and support to clinical colleagues in the medical wards, following referral of patients. Further integration will require attending to other PC needs in the hospital such as the lack of trained staff, research, SOPs across the hospital and providing all staff with tools and training to manage moral injury when working with patients who are suffering.

This moral distress experienced by HCPs is a tangible but unspoken factor in ATH. It is recognized in this study and other studies that without the skills to care for patients with life-threatening illnesses, there is over-investigation, avoidance and even hopelessness among HCPs.^19^ Sallnow *et al.* states that conversations about death and dying are difficult and avoidance is preferred. A result is continuation of inappropriate treatments, which inevitably leads to a high-cost medicalized end-of-life care package for patients.^19^ PC services and education are thus fundamental in ensuring the non-abandonment and appropriate treatment of patients with life-limiting illnesses, cost-effective care and addressing distress in HCPs, patients and families.

### PC education

However, the urgency around PC education is more complex than the global and national call for PC education.^1,2^ Unfortunately, PC is still associated with only end-of-life care, a topic many HCPs, including educators, avoid.^19,43^ The research participants relate this avoidance to the complexities of HCPs facing their own mortality. Besides the emotive nature of PC education, PC is not an academic requirement in many disciplines, reinforcing misconceptions, avoidance, discomfort and ultimately limited PC provision. In an overstretched existing curriculum, PC education is not valued as an essential component of training. Refraining from integrating PC education into all medical and nursing curricula is cemented by the assumption that HCPs are already providing PC and that further education is not required, as discovered by this research. Although national and international policies highlight the need for a PC-trained healthcare force and recognize that additional training is required, especially in specialities who work daily with patients with life-limiting illnesses, PC has not been voluntarily integrated into training curricula.^2,32^ Sustainable and recommended PC education requires mandatory PC education integration across curricula as indicated by global standards.^2,44^ These assumptions play out beyond the educational paradigm, including hospital and clinical leadership and may also require further mandatory interventions.

### The gap in clinical leadership for PC and accountability structures

Many senior clinical leaders have received little to no PC training in South Africa.^45,46^ These individuals are the clinical decision-makers and leaders in the wards. Junior PC-trained colleagues have recognized that it is difficult to initiate a PC approach when the decision-makers do not support this approach.^47^ Clinical leadership is a recognized functional dimension to enable clinical integration.^21^ This lack of senior clinical leadership has a ripple effect beyond immediate clinical care as it results in absence from clinical guidelines as well as inhibiting the transformational effect of PC that is envisioned by international and national policies. South Africa has only recently recognized PC as a subspeciality, which impedes clinical leadership. A consequence, as demonstrated in this study, is that PC-related governance, training and information systems do not exist in all the departments, and many disciplines steer away from taking ownership of their PC service delivery. The reluctance of clinical leaders to embrace the PC approach may be a function of the misconception that it is only for dying patients. Traditionally, a dying patient is linked with poor outcomes and perhaps even poor care. In a hospital striving for excellence, poor outcomes are not welcomed.^27^ Therefore, if PC outcomes are not measured as an indicator of good care, the need to strive for good PC outcomes may seem unnecessary and counterproductive. Including and measuring PC outcomes as measures of excellence may strengthen accountability towards patients and families with life-threatening illnesses.

Accountability structures are not included in the National Policy and the Western Cape Model for Implementation.^1,36^ This study also found that the main guidelines, policies and management structures were created in the vertical nurse-driven service, with limited evidence of further integration in the ATH. From the above it is evident that the PC integration is being driven by a small group of people within the PC service and the university which is not fully integrated across the hospital. This finding is concerning as the long-term sustainability of PC is in jeopardy if it is driven by only a small group of individuals.^48^

### Siloed approach a barrier to integration

This lack of integration is not just a phenomenon experienced in PC because the hospital functions in silos, and HCPs stay focused on their discipline. Services are linked rather than integrated in the hospital.^49^ In South African ATHs, different disciplines are siloed to manage a high volume of patients with limited resources. Moreover, these patients have complex disease profiles. As the preferred method of service delivery, stakeholders may not willingly change.^49^ Therefore, aiming to provide horizontal care may not align with the ‘way things are done’ in the ATH’s prevailing vertical delivery system.^50^ Changing a prevailing organizational culture is labelled as a ‘major’ change that may be beyond a new emerging intervention.^50^ Furthermore, PC is not aligned with a specific academic discipline or the disease approach to care. The national recognition of PC as subspecialized care with specific skills and role is a necessary and fundamental global indicator that reflects PC development in a country.^44^ PC in an ATH will, therefore, have to create its own unique ‘silo’ to align with the way the hospital functions and with global PC development to ensure a PC focus is maintained. Furthermore, PC cannot be confined to one level of care.

The alignment problem in PC is also evident in the district services, which indirectly impedes PC status and integration in the ATH. This research has demonstrated that managers value bed turnaround time because it is essential to ensure that as many patients as possible receive care with curative intent in ATH. Thus discharging patients to functional PC services in the district is fundamental to valuing PC. Patients who require long-term PC may be ‘blocking beds’ in ATH and would probably receive better and more appropriate care in their own homes or hospice beds.^19^ The high cost, over-medicalized care is known to be a negative consequence of hospitalized end-of-life care.^19^ Unfortunately, PC is not fully integrated into district services, and there are still limited dedicated PC beds in district services with limited trained home PC providers. Whole system oblique integration, especially into communities, is thus core to the integration in ATHs to ensure patients receive excellent care and the appropriate care at the right time.^41^

### PC diplomacy

Despite these challenges, GSH has achieved a level of PC integration. This service is supported by the university clinically and also in relationship-building outside and inside the hospital. It is linked to many hours of supporting HCPs with clinical care and sometimes assisting HCPs to cope with their own distress. PC diplomacy and steadfast relationships are required to build trust within the healthcare system. Factors such as being of service, being always available, being invited in, being grateful for receiving referrals, being modest and being transparent are recognized normative factors that enable integration in PC.^51^ With limited resources, diplomacy may be core to pushing the PC agenda. However, this service will require further support, especially due to the multifaceted PC need, if we are to prevent burnout in existing services. In addition, without established PC services in ATHs there will not be sufficient research, educational and clinical support to drive national PC integration.

## Conclusion

This research has highlighted the interplay between micro, meso and macro level in the integration of PC in one ATH in South Africa. This is further impacted by the interplay between functional and the normative aspects in the South African ATH context that is filled with misconceptions regarding PC, competing values, resource limitations, vertical structures and lack of PC accountability structures. Therefore, mandatory PC education, national recognition of the specialized nature of PC, national and local monitoring and evaluation, standards and guidelines and oblique integration across the health system are fundamental in PC integration but are currently not in place.

Understanding the interconnectedness of the above-mentioned normative and functional aspects in the context of PC integration may assist in developing strategies to break this repetitive negative feedback. Furthermore, understanding the specific clinical settings’ context may assist in developing strategic interventions when integrating PC. This may in turn, assist in the transferability of these strategies to other settings.

## Limitations

This research is situated within GSH, a relatively well-functioning ATH in the South African setting with many years of PC sensitization. This may not be true for other ATHs in South Africa but the approach used to describe the context does provide an analytical approach for other settings. GSH is also within a province that has already launched a PC policy, and that has relatively stable primary care and secondary care. GSH is also linked to hospices across the WC, contributing to the continuity of care. This may influence the generalizability within the South African context, especially in areas without well-established referral pathways.

Most of the data was obtained through interviews. Participants may not have given a true reflection of actual events and reasons for integrating PC to prevent negative impressions.

## Declarations

**Ethics approval and consent to participate:** Ethics approval was obtained from the University of Cape Town Human Ethics Committee (HREC 071/2020) and site permission was obtained from Groote Schuur Hospital. The REDCap database also has ethics approval from the UCT Human Ethics Committee (HREC R012/2016). Informed consent was obtained from the interviewees and patients or their families on the database. All consented to their data being included. All data were anonymized, stored on a password-protected data base with secure access.

**Consent for publication:** Consent to publication was obtained from participants.

**Availability of data and materials:** Anonymized data is available from the corresponding author on reasonable request.
